# Evaluation of the brain hemodynamic response by means of near-infrared spectroscopy (NIRS) monitoring in patients with atherosclerotic carotid disease undergoing endarterectomy

**DOI:** 10.1590/1677-5449.190027

**Published:** 2020-03-06

**Authors:** Letícia Cristina Dalledone Siqueira Rein, Daniel Emílio Dalledone Siqueira, Ana Terezinha Guillaumon, Wagner Mauad Avelar, Fernando Cendes, Rickson Coelho Mesquita

**Affiliations:** 1 Universidade Estadual de Campinas – UNICAMP, Faculdade de Ciências Médicas, Campinas, SP, Brasil.

**Keywords:** carotid artery disease, carotid stenosis, carotid endarterectomy, near-infrared spectroscopy

## Abstract

**Backgrounds:**

Near-infrared spectroscopy (NIRS) is non-invasive technique that detects hemodynamic alterations in tissues. It enables continuous monitoring of intracerebral vascular physiologic information. Due to its portable nature, NIRS may be used bedside or in the operating room.

**Objectives:**

To evaluate use of NIRS for intraoperative monitoring of the brain hemodynamic response, during carotid endarterectomy.

**Methods:**

10 patients with atherosclerotic carotid disease scheduled for endarterectomy were evaluated. After patients had been selected, they answered a questionnaire on epidemiological data and information about comorbidities and then carotid disease was confirmed with diagnostic methods. NRIS monitoring was used during the surgical procedure. The variables analyzed before, during and after carotid clamping were oxygen saturation (SatO_2_), total hemoglobin (THb), reduced hemoglobin (RHb), and oxyhemoglobin (OHb). A p value of <0.05 was considered statistically significant.

**Results:**

The results obtained from NIRS show that RHb and SatO_2_ vary during the different stages of surgery. RHb levels are higher during clamping, when compared with the other two surgical stages. On the other hand, SatO_2_ is lower during clamping.

**Conclusions:**

During carotid endarterectomy, NIRS is a feasible, real-time, and non-invasive intracranial monitoring method that accurately and reliably measures the changes in intracerebral capillary hemodynamic conditions.

## INTRODUCTION

Carotid endarterectomy is a useful procedure for prevention of later stroke episodes in patients with severe lesions of the common and internal carotid arteries. Reducing the morbidity and mortality associated with these procedures is fundamental to the method’s safety and viability.^1^

There is a growing trend to use methods during carotid artery endarterectomy that are capable of providing information on neurometabolic status, of assessing residual lesions and technical defects, and, primarily, of analyzing cerebral conditions, avoiding postoperative neurological deficits.^2^

Near-infrared spectroscopy (NIRS) is a noninvasive technique that used portable and easily-handled equipment and is capable of providing information about cerebral hemodynamic conditions, having most of the characteristics of an ideal method.

Near-infrared spectroscopy uses the infrared region of the electromagnetic spectrum (600 to 900 nanometers) to measure oxygen concentrations. Near infrared is the name given to the region immediately above the visible region, i.e., it is the part of the infrared region that is closest to the visible region. Applications for these wavelengths have been increasing over recent decades.

## OBJECTIVES

The objective of this study was to demonstrate the initial experience of the vascular diseases team at the Universidade Estadual de Campinas (UNICAMP) Department of Surgery with use of near-infrared spectroscopy (NIRS) for intraoperative monitoring of cerebral hemodynamic response during endarterectomy of carotid arteries with atherosclerotic disease, analyzing the behavior of variables that indicate the hemodynamic responses (total hemoglobin, oxyhemoglobin, reduced hemoglobin, and oxygen saturation), measured using NIRS at three points during carotid artery endarterectomy: before clamping, during clamping, and after clamping.

## METHODS

This study was approved by the Research Ethics Committee at the Universidade Estadual de Campinas (CAAE: 09911113.2.0000.5404) and authorized by the teaching, research, and extension center at the University Hospital where the study was conducted.

This is a prospective, cross-sectional, clinical cohort study. It was conducted in the vascular diseases sector of the Surgery Department of the Unicamp Medical Sciences Faculty, at the Hospital de Clínicas da Unicamp.

The study sample comprised 10 people over the age of 50, of both sexes, with carotid artery disease of atherosclerotic etiology, previously detected because of clinical manifestations (transient ischemic attack or stroke) and confirmed by arterial Doppler ultrasound of the carotids and arterial computed angiotomography of the supra-aortic trunks. Consecutive patients were recruited who had indications for carotid artery endarterectomy and agreed to take part, signing free and informed consent forms.

Carotid artery endarterectomy was indicated for patients with unilateral carotid stenosis greater than or equal to 70%. Patients underwent carotid endarterectomy with NIRS monitoring throughout the entire surgical procedure before, after, and during clamping. After the procedure, patients were followed up at the vascular diseases clinic, in accordance with its routine protocol, with periodic outpatients follow-up and assessment with Doppler ultrasound.

Patients were excluded from the sample if they had progressive stroke still in course, with progressive symptomology, were asymptomatic with carotid stenosis detected by routine imaging exams, or if, for whatever reason, they refused to participate in any of the stages of the study.

Carotid artery endarterectomy was conducted using the classic technique, without carotid shunt and under general anesthesia. All patients were monitored intraoperatively with NIRS, including the entire periods before, during, and after clamping. Additionally, control arterial blood gas analysis was conducted for all three surgical phases.

Near-infrared spectroscopy was conducted using a commercial FD-DOS (diffusion optical spectroscopy) system (Imagent, IIS Inc., United States) for data capture. This system employs the optrodes reflectively (in that both are on the same side of the surface) with a continuous illumination excitation method. The system comprises a photomultiplier tube, as detector, and four diode lasers, as light sources, with a modulation frequency of 110 mhz. Each source has a different wavelength, from 690 to 840 nm.

**Figure 1:**
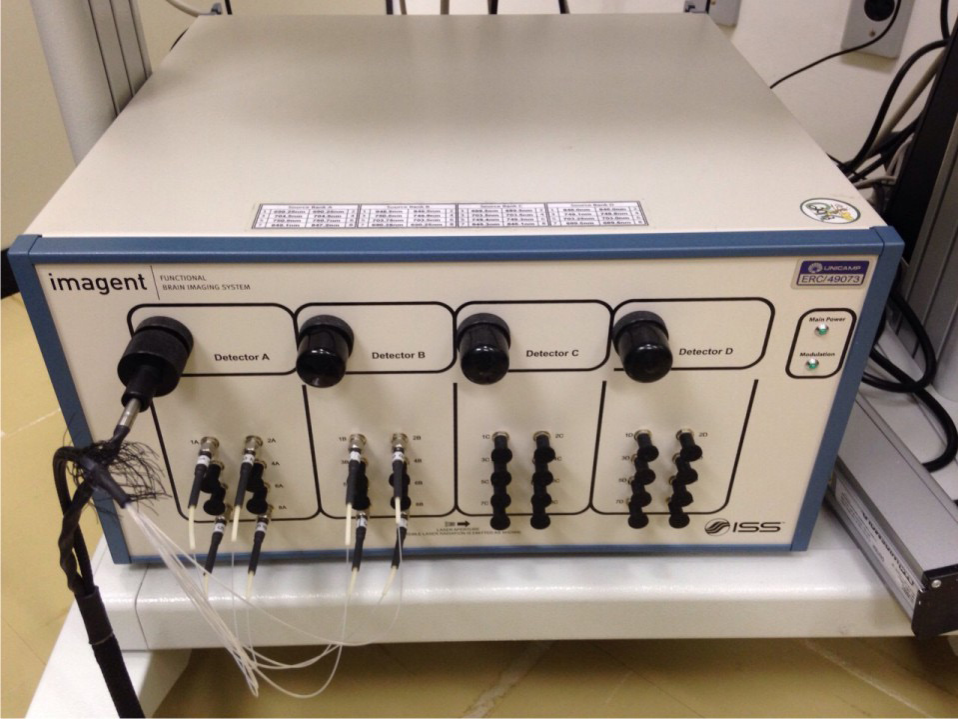
The NIRS machine.

**Figure 2:**
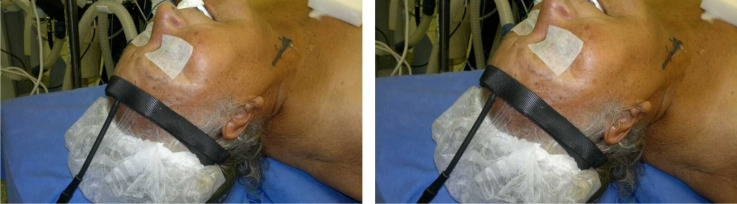
Placement of the NIRS optical probe.

The optical probe was positioned over the prefrontal cortex on the ipsilateral side to the stenosis to monitor cerebral circulation (Figures 1 and 2).

The variables analyzed with NIRS were: total hemoglobin, reduced hemoglobin, oxyhemoglobin, and oxygen saturation. All variables were recorded at 0.05 second intervals. After data had been collected, a database was constructed for statistical analysis and to plot figures and populate tables.

Initially, epidemiological data on the patients were analyzed descriptively, calculating certain summary measures, such as means, medians, ranges, standard deviations, counts, and percentages.

Next, to summarize the information on the patients obtained with NIRS during each phase of surgery, a third degree polynomial was fitted to the data and the coefficients obtained were used as summary measures for each variable in each experimental situation.

The Friedman test and the Bonferroni multiple comparisons method were used for inferential analysis to compare the variables of interest across each of the three phases of surgery.

The conclusions of the statistical analysis were drawn from the inferential analyses, with a significance level of p = 5%.

## RESULTS

The sample selected for the study comprised10 patients who were treated with carotid artery endarterectomy surgery using the classic technique. There were three women and seven men, with a mean age of 70.7 years, varying from 56 to 79 years, with a standard deviation of 7.4 years. The most common comorbidities were smoking, systemic arterial hypertension, and dyslipidemia. The Goldman index was used for cardiological risk stratification and the Torrington scale was used for pulmonary risk.

The mean duration of carotid endarterectomy surgery was 106.8 minutes, ranging from 68 to 150 minutes, with a standard deviation of 33.1 minutes. In terms of side of surgical procedures, 70% were on the right and 30% on the left. The minimum degree of stenosis of the internal carotid artery for surgery to be indicated was 70%. None of the patients had disease involving the vertebral arteries and in all patients the circle of Willis was patent.

All patients underwent carotid clamping, with mean duration of 22 minutes and standard deviation of 10.5 minutes. There were no neurological complications during or after the operations.

Arterial blood gas analysis was performed at three distinct phases of the surgical procedure, before, during, and after clamping, measuring pH, partial O_2_ pressure, partial CO_2_ pressure, oxygen saturation (SatO_2_), lactate, hematocrit, and hemoglobin level.

**Figure 3:**
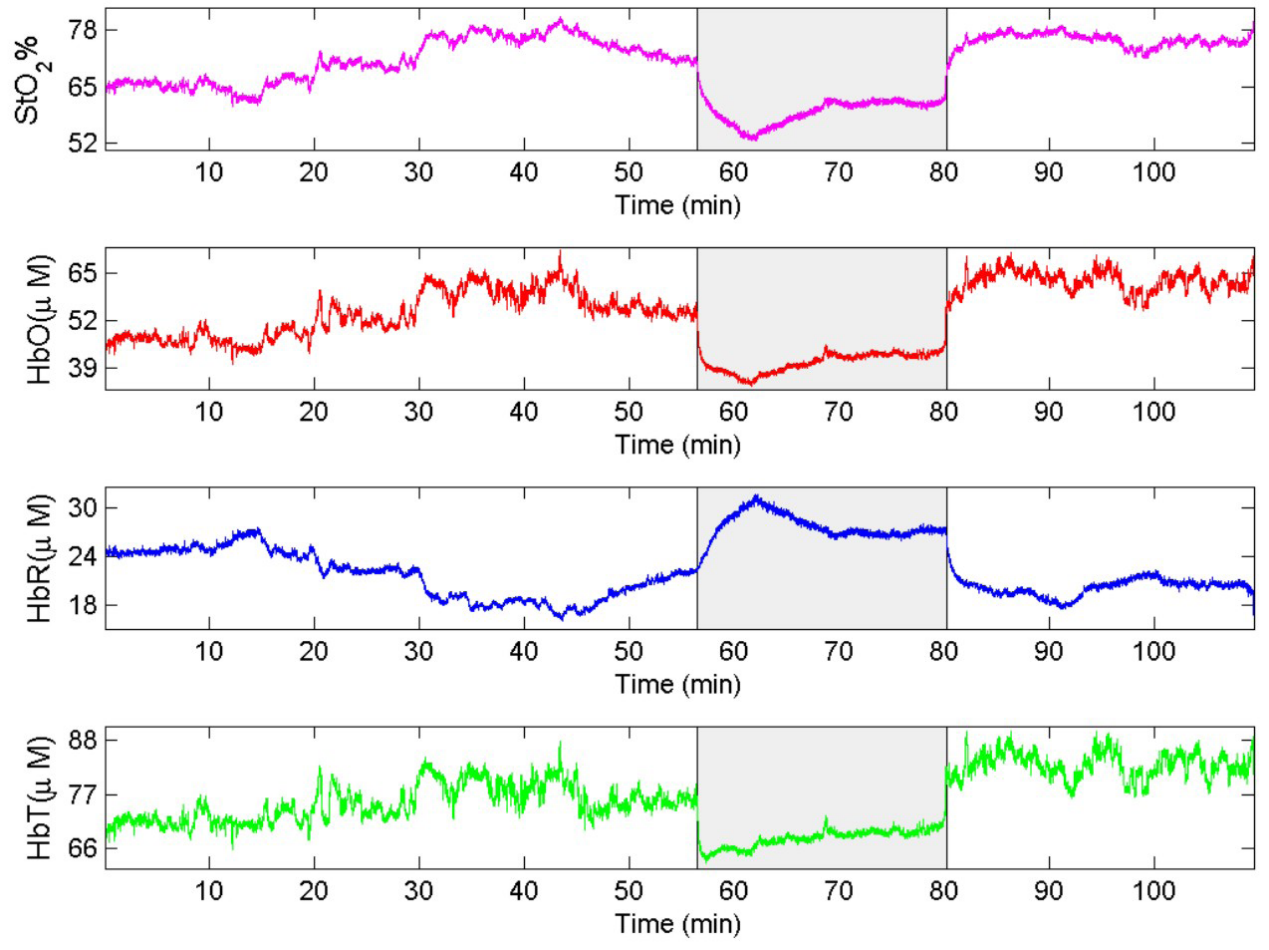
Graph obtained from NIRS monitoring of volunteer 9, showing, on the x-axis, time in minutes and, on the y-axis, oxygen saturation (SatO_2_; %) in magenta, oxyhemoglobin (HbO; micro mol) in red, reduced hemoglobin (HbR; micro mol) in blue, and total hemoglobin (HbT; micro mol) in green; the shaded area indicates clamping.

Analysis of the results obtained from the measurements recorded by the NIRS system supports the conclusion that the three stages of surgery differ in terms of the variables HbR and SatO_2,_ as shown in Tables 1, 2, and 3. During clamping, the values of the HbR variable were higher than during the other two phases of surgery. In contrast, the SatO_2_ variable reduced during clamping, as illustrated in Figure 3. This graph, obtained from NIRS monitoring of volunteer 9, shows, on the x-axis, time in minutes and, on the y-axis, oxygen saturation (SatO_2_; %) in magenta, oxyhemoglobin (HbO; micro mol) in red, reduced hemoglobin (HbR; micro mol) in blue, and total hemoglobin (HbT; micro mol) in green; the shaded area indicates clamping.

**Table 1:** Descriptive levels obtained by comparison of the three phases of surgery for the summary measures of the study.

**Variable**	**Coefficient 1**	**Coefficient 2**	**Coefficient 3**
HbO	0.150	0.301	0.301
HbR	0.007	0.007	0.007
HbT	0.211	0.202	0.670
SatO_2_	0.014	0.045	0.045

HbO = oxyhemoglobin ; HbR = reduced hemoglobin; HbT = total hemoglobin; SatO_2_ = oxygen saturation.

**Table 2:** Descriptive levels obtained from two-by-two comparisons of phases, for the variable reduced hemoglobin (HbR).

**Phases compared**		**Coefficient 1**	**Coefficient 2**	**Coefficient 3**
Before clamping	Clamped	0.029	0.029	0.029
Before clamping	After clamping	0.064	0.084	0.131
Clamped	After clamping	0.018	0.016	0.016

**Table 3:** Descriptive levels obtained from two-by-two comparisons of phases, for the variable oxygen saturation (SatO_2_).

**Phases compared**		**Coefficient 1**	**Coefficient 2**	**Coefficient 3**
Before clamping	Clamped	0.131	0.160	0.322
Before clamping	After clamping	0.021	0.029	0.046
Clamped	After clamping	0.016	0.029	0.046

**Figure 4:**
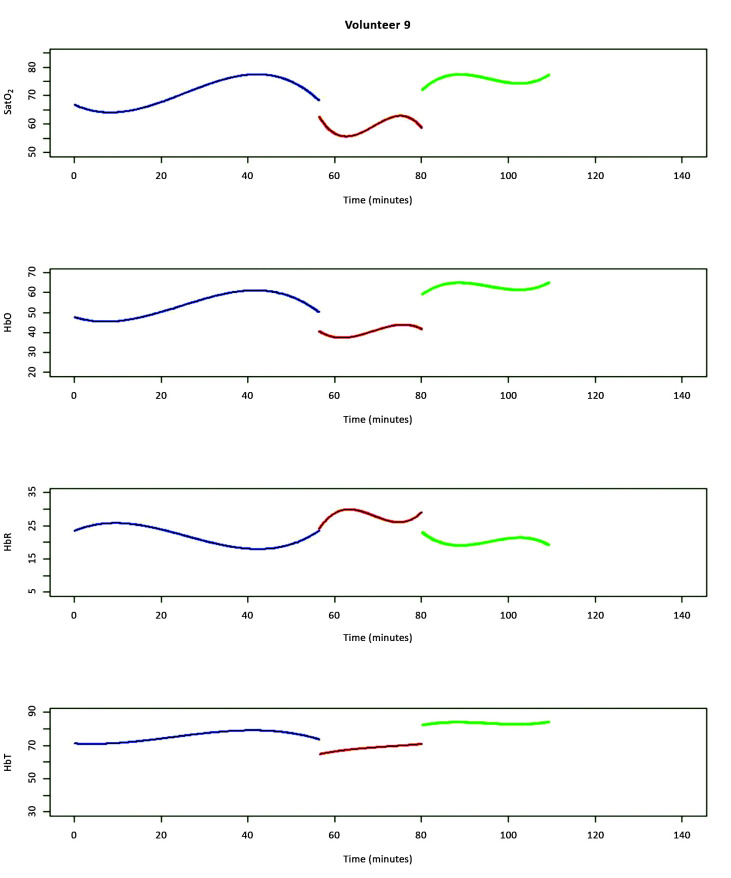
Fitted polynomials for volunteer 9, where the “before clamping” phase is in blue, the “during clamping” phase is in red, and the “after clamping” phase is in green, for each of the four variables employed (SatO_2_, HbO, HbR, and HbT).

An example graph (Figure 4) from one of the volunteers shows the fitted polynomials, where the “before clamping” phase is in blue, the “during clamping” phase is in red, and the “after clamping” phase is in green, for each of the four variables employed (SatO_2_, HbO, HbR, and HbT).

## DISCUSSION

Since the 1980s, many studies have been published reporting the utility of near infrared for monitoring tissue oxygenation under diverse clinical conditions. However, to date, no studies have correlated an application of near infrared during carotid artery endarterectomy, by means of continuous monitoring before, during and after carotid clamping, and this paper is the first to discuss this subject.

In theoretical and practical terms, Murkin and Arango used NIRS to detect ischemia in other territories, for example, splanchnic, renal, and vertebral. This explains the sensitivity and clinical applicability of this method for continuous monitoring of cerebral perfusion by NIRS to detect changes caused by surgical treatment in the carotid territory.^3^

According to Becquemin et al., there are certain criteria to determine the choice of the surgical method to be employed with patients with carotid stenosis. Presence of coronary disease, aortoiliac disease, or renal failure, age (over 80 years), and history of cancer (possible treatment with radiotherapy) are examples of some of the risk factors that have a direct influence on whether carotid artery endarterectomy or carotid angioplasty with stenting should be used.^4^

All of the patients in our sample were monitored intraoperatively using pulse oximetry and capnography, demonstrating constant stability of these parameters during the surgical procedure.

Many different cerebral monitoring methods have been described in the literature, but there is no method that can safely and effectively show ischemic cerebral changes. Therefore, there is a constant search for safe and reproducible methods that can reduce the morbidity and mortality of surgery. Sloan states that during vascular surgery in the carotid territory and cardiovascular surgery, neuroimaging techniques provide important information that can be seen by those conducting the surgical techniques and, possibly, improve clinical results. However, these techniques are imperfect and diagnostic methods have not yet been precisely established.^5^

Use of NIRS appears to be a promising option for the near future, but wider-ranging clinical trials will be needed to investigate the many different areas involved in tissue ischemia. Fellahi et al. have stated that oxygen saturation values are different in different vascular beds and that there is a difference in tolerance of ischemia between men and women.^6^

The underlying principals of NIRS analysis lie in application of different wavelengths, leading to qualitative and quantitative differences between the molecular components of a biological tissue. The method of capture is dependent on the effects of reflection, diffusion, and absorption.

There is a wide range of applications for NIRS. One example can be found in a study by Casati et al., where monitoring was important for guiding anesthesia planning in elderly patients undergoing abdominal surgery, reducing exposure to ischemia, with reduced cognitive effects and shorter hospital stay. Many clinical conditions seen in daily medical practice have the potential to cause changes to cerebral oxygenation, leading to a risk of intraoperative cerebral ischemia. Identification of these changes to the cerebral oxygenation equilibrium with a simple and effective method has the potential to optimize anesthetic planning to meet each person’s true requirements for the most important organ, the brain.^7^

In our sample, we observed that the measurements recorded by NIRS revealed differences in the variables HbR and SatO_2_ during the phases before and after clamping, in comparison with the clamping phase. This constitutes cerebral tissue ischemia detected by the method in a direct manner, showing reductions in tissue oxygen and increases in CO_2_ levels.

In 2004, Mille et al.^8^ conducted a study of NIRS monitoring during carotid endarterectomy with the objective of determining which patients had good collateralization of the cerebral circulation during clamping of the carotid, by identifying the percentage decrease in local oxygen saturation. They suggested that when the decrease in oxygen saturation in relation to the baseline value before clamping is less than or equal to 20%, ischemia and hypoperfusion are infrequent and shunting is unnecessary. Decreases greater than 20% do not always indicate an intraoperative neurological complication, but can be used to define conduct.

In 2007, Yamamoto et al.,^9^ stated that hypoperfusion was one of the factors that lead to stroke during the perioperative period of carotid endarterectomy. Selective shunting requires simple and sensitive monitoring. According to these authors, NIRS is a monitoring system that could be used during surgery and one that instantly reflects oxygenation.

The NIRS method and its mode of application are novel, which explains the inexperience of the team, who decided to report their initial experience and, in later studies that are already ongoing, expand the sample.

## CONCLUSIONS

Near infrared spectroscopy (NIRS) is a feasible and applicable method for noninvasive intracerebral monitoring in real time during carotid endarterectomy.

This technique is capable of measuring changes in the levels of oxygen saturation, total hemoglobin, reduced hemoglobin, and oxyhemoglobin during the three phases of carotid endarterectomy (before, during, and after clamping).
