# Supplementary material for: Evaluation of the brain hemodynamic response by means of near-infrared spectroscopy (NIRS) monitoring in patients with atherosclerotic carotid disease undergoing endarterectomy
Source: J Vasc Bras. 2020 Mar 6;19:e20190027. doi: 10.1590/1677-5449.190027 (PMC8205114; doi:10.1590/1677-5449.190027)
Supplement: Translation

# Translation: Avaliação da resposta hemodinâmica cerebral através da monitorização com a espectroscopia próxima ao infravermelho (NIRS) em pacientes com doença aterosclerótica da artéria carótida submetidos a endarterectomia

**Authors:** Letícia Cristina Dalledone Siqueira Rein, Daniel Emílio Dalledone Siqueira, Ana Terezinha Guillaumon, Wagner Mauad Avelar, Fernando Cendes, Rickson Coelho Mesquita

**Affiliations:** 1 Universidade Estadual de Campinas – UNICAMP, Faculdade de Ciências Médicas, Campinas, SP, Brasil.

**Keywords:** doenças das artérias carótidas, endarterectomia de carótidas, estenose das carótidas, espectroscopia de luz próxima ao infravermelho

## Abstract

**Contexto:**

A espectroscopia próxima ao infravermelho (NIRS) é uma técnica não invasiva que detecta as alterações hemodinâmicas teciduais. A NIRS pode monitorar de forma contínua as informações fisiológicas vasculares intracranianas. Por ser portátil, ela pode ser utilizada à beira do leito e no centro cirúrgico.

**Objetivos:**

Avaliar as possíveis alterações hemodinâmicas cerebrais durante a endarterectomia em pacientes com estenoses maiores que 70% utilizando NIRS.

**Métodos:**

Foram avaliados 10 voluntários portadores de doença carotídea aterosclerótica com indicação de endarterectomia. Após a seleção dos pacientes, que responderam um questionário com dados epidemiológicos e informações referentes à presença de comorbidades, a doença foi confirmada por métodos diagnósticos. No procedimento cirúrgico, utilizou-se a NIRS para monitorização. Foram avaliadas as variáveis saturação de oxigênio (SatO_2_), hemoglobina total (HbT), hemoglobina reduzida (HbR) e hemoglobina oxigenada (HbO) nos três tempos cirúrgicos pré-, trans e pós-clampeamento carotídeo. Utilizou-se p < 0,05 como nível de significância.

**Resultados:**

A avaliação dos resultados obtidos por meio das medidas registradas pela NIRS permite afirmar que HbR e SatO_2_ variam ao longo das etapas da cirurgia. Durante o clampeamento, a variável HbR mostra valores mais elevados que nas outras duas etapas da cirurgia. Por outro lado, a variável SatO_2_ mostra redução durante o clampeamento.

**Conclusões:**

A NIRS é um método viável e aplicável de monitorização intracerebral, não invasivo e em tempo real, durante a endarterectomia carotídea, capaz de medir de forma precisa as mudanças das condições hemodinâmicas capilares intracerebrais.

## INTRODUÇÃO

A endarterectomia carotídea é um procedimento útil para a prevenção de episódios subsequentes de acidentes isquêmicos cerebrais em pacientes com lesões severas em artéria carótida comum e interna. Diminuir a morbimortalidade desses procedimentos é fundamental para a segurança e viabilidade do método^1^.

É crescente a utilização de métodos que, durante a endarterectomia de artéria carótida, forneçam informações do estado neurometabólico e avaliem lesões residuais, defeitos técnicos e, principalmente, analisem a condição cerebral, evitando déficits neurológicos no pós-operatório^2^.

A espectroscopia próxima ao infravermelho (NIRS) é uma técnica não invasiva, portátil e de fácil manuseio, capaz de fornecer as informações sobre as condições hemodinâmicas cerebrais e que contempla a maioria das características de um método ideal.

A NIRS é um método que utiliza a região infravermelha do espectro eletromagnético, 600 a 900 nanômetros, para medir a concentração de oxigênio. O infravermelho próximo é o nome dado à região imediatamente superior à região visível, em termos de comprimento de onda, ou seja, trata-se da região do infravermelho mais próxima da região visível. Seu uso vem crescendo nas últimas décadas.

## OBJETIVOS

Os objetivos da pesquisa foram demonstrar a experiência inicial da disciplina de Moléstias Vasculares do Departamento de Cirurgia da Universidade Estadual de Campinas (UNICAMP) na utilização da espectroscopia próxima ao infravermelho (NIRS) para monitorização intraoperatória da resposta hemodinâmica cerebral durante a endarterectomia de artéria carótida acometida por doença aterosclerótica.

Analisar o comportamento das variáveis que avaliaram as respostas hemodinâmicas (hemoglobina total, hemoglobina oxigenada, hemoglobobina reduzida e saturação de oxigênio) obtidas com a utilização da NIRS nos três momentos da endarterectomia de artéria carótida: pré-clampeamento, transclampeamento e pós-clampeamento.

## MÉTODOS

Este estudo foi aprovado pelo Comitê de Ética em Pesquisa da Universidade Estadual de Campinas (CAAE: 09911113.2.0000.5404) e autorizado pelo Núcleo de Ensino, Pesquisa e Extensão do Hospital Universitário em que foi realizado.

Trata-se de um estudo clínico de coorte, transversal e prospectivo. O estudo foi realizado na disciplina de Moléstias Vasculares do Departamento de Cirurgia da Faculdade de Ciências Médicas da Unicamp, no Hospital de Clínicas da Unicamp.

A amostra do estudo foi composta por 10 indivíduos, de ambos os sexos, com idade superior a 50 anos, portadores de doença arterial carotídea de etiologia aterosclerótica, previamente detectada por meio de manifestações clínicas (ataque isquêmico transitório ou acidente vascular encefálico), confirmada por meio de ultrassom doppler arterial de carótidas e angitomografia computadorizada arterial de troncos supra-aórticos. Foram incluídos na pesquisa os indivíduos, de maneira consecutiva e com indicação cirúrgica de endarterectomia de artéria carótida, que concordaram em participar e assinaram o termo de consentimento livre e esclarecido (TCLE).

Foi indicada endarterectomia de artéria carótida em doentes com estenose carotídea unilateral maior ou igual a 70%. Os pacientes foram submetidos à endarterectomia de carótida com monitorização pela NIRS, durante todo o procedimento cirúrgico pré-, pós- e transclampeamento. Após o procedimento, os pacientes prosseguiram seu acompanhamento no ambulatório da disciplina, conforme protocolo da disciplina, com retorno ambulatorial periódico e avaliação com ultrassom Doppler.

Foram excluídos da amostra os indivíduos com acidente vascular cerebral (AVC) em evolução com sintomatologia progressiva, indivíduos assintomáticos com estenose carotídea detectada por meio de exames de imagem rotineiros e indivíduos que, por algum motivo, recusaram-se a participar de qualquer uma das etapas do estudo.

A endarterectomia de artéria carótida foi realizada utilizando a técnica clássica, sem shunt carotídeo e sob anestesia geral. Todos os pacientes foram monitorizados no intraoperatório com NIRS, incluindo a totalidade dos períodos pré-, trans e pós-clampeamento. Além disso, procedeu-se a realização de gasometria arterial de controle nos três tempos cirúrgicos.

A NIRS foi realizada por meio do sistema FD-DOS (espectroscopia óptica de difusão) comercial (Imagent, IIS Inc., EUA) para aquisição dos dados. Trata-se de um aparelho que utiliza os optodos de forma reflexiva (em que ambos ficam do mesmo lado da superfície), em que o método de excitação é a iluminação contínua. O sistema é composto por um tubo fotomultiplicador como detector e quatro lasers de diodo como fontes de luz com uma frequência de modulação de 110 mhz. Cada fonte tem diferentes comprimentos de onda, de 690 a 840 nm.

A sonda óptica foi posicionada sobre o córtex pré-frontal no lado ipsilateral à estenose, para monitoramento da circulação cerebral (Figuras 1 e 2).

**Figura 1:** Detalhe do aparelho de NIRS.

**Figura 2:** Posicionamento da sonda óptica da NIRS.

As variáveis analisadas pela NIRS foram: hemoglobina total, hemoglobina reduzida, hemoglobina oxigenada e saturação de oxigênio. Todas as variáveis foram registradas em intervalos de 0,05 segundos cada. A partir da coleta das informações, foi montado um banco de dados para análise estatística e confecção de figuras e tabelas.

Inicialmente, considerando os dados epidemiológicos dos pacientes, a análise foi feita de forma descritiva por meio do cálculo de algumas medidas-resumo, como média, mediana, valores mínimo e máximo, desvio-padrão, frequências absoluta e relativa (porcentagem).

Posteriormente, para resumir as informações obtidas com a NIRS dos pacientes em cada etapa da cirurgia, ajustou-se um polinômio de grau 3 aos dados, e os coeficientes obtidos foram empregados como medidas-resumo para cada variável e em cada situação experimental.

Para a análise inferencial para comparar as três etapas da cirurgia quanto a cada uma das variáveis de interesse, considerando-se as medidas-resumo, empregou-se o teste de Friedman e o método de comparações múltiplas de Bonferroni.

Na análise estatística, as conclusões foram obtidas por meio das análises inferenciais utilizando o nível de significância p igual a 5%.

## RESULTADOS

A amostra selecionada da pesquisa foi composta por 10 doentes, que foram submetidos a cirurgia de endarterectomia de artéria carótida pela técnica clássica, sendo três mulheres e sete homens, com média de idade de 70,7 anos, variando de 56 a 79 anos, com desvio-padrão de 7,4 anos. Entre as comorbidades mais frequentes observou-se tabagismo, hipertensão arterial sistêmica e dislipidemia. Quanto à estratificação de risco cardiológico, utilizou-se o Índice de Goldmann; e quanto ao risco pneumológico, o Índice de Torrington.

O tempo médio de duração da cirurgia de endarterectomia de carótida foi de 106,8 minutos, variando de 68 a 150 minutos, com desvio-padrão de 33,1 minutos. Quanto à lateralidade da amostra, dos procedimentos cirúrgicos, o resultado foi 70% à direita e 30% à esquerda. O grau de estenose mínimo da artéria carótida interna para indicação da cirurgia foi de 70%. Nenhum paciente apresentava doença nas artérias vertebrais, e todos apresentavam polígono de Willis pérvio.

Todos os pacientes foram submetidos a clampeamento carotídeo, com duração média de 22 minutos e desvio-padrão de 10,5 minutos. Não houve complicações neurológicas no intra e no pós-operatório.

Quanto à análise da gasometria arterial, em três momentos distintos do procedimento cirúrgico: pré, trans e pós-clampeamento, foram analisados potencial hidrogênionico (pH), pressão parcial de O_2_, pressão parcial de CO_2_, saturação de oxigênio (SatO_2_), lactato, hematócrito e nível de hemoglobina.

A avaliação dos resultados obtidos por meio das medidas registradas pela NIRS permite afirmar que as etapas da cirurgia diferem quanto às variáveis HbR e SatO_2,_ conforme demonstrado nas Tabelas 1, 2 e 3. Durante a etapa do clampeamento, a variável HbR mostra valores mais elevados que nas outras duas etapas da cirurgia. De outra parte, a variável SatO_2_ mostra redução durante o clampeamento, conforme a Figura 3. Gráfico obtido com a monitorização por NIRS no voluntário 9, demonstrando, no eixo x, o tempo em minutos e, no eixo y, a saturação de oxigênio (SatO_2_; %) em lilás, a hemoglobina oxigenada (HbO; micro mol) em vermelho, a hemoglobina reduzida (HbR; micro mol) em azul e a hemoglobina total (HbT; micro mol) em verde; a área sombreada corresponde ao clampeamento.

**Tabela 1:** Níveis descritivos obtidos da comparação entre as três etapas da cirurgia, quanto às medidas-resumo do estudo.

**Variável**	**Coeficiente 1**	**Coeficiente 2**	**Coeficiente 3**
HbO	0,150	0,301	0,301
HbR	0,007	0,007	0,007
HbT	0,211	0,202	0,670
SatO_2_	0,014	0,045	0,045

HbO = hemoglobina oxigenada; HbR = hemoglobina reduzida; HbT = hemoglobina total; SatO_2_ = saturação de oxigênio.

**Tabela 2:** Níveis descritivos obtidos da comparação entre as etapas duas a duas, quanto à variável hemoglobina reduzida (HbR).

**Etapas comparadas**		**Coeficiente 1**	**Coeficiente 2**	**Coeficiente 3**
Pré-clampeamento	Clampeamento	0,029	0,029	0,029
Pré-clampeamento	Pós-clampeamento	0,064	0,084	0,131
Clampeamento	Pós-clampeamento	0,018	0,016	0,016

**Tabela 3:** Níveis descritivos obtidos da comparação entre as etapas duas a duas, quanto à variável saturação de oxigênio (SatO_2_).

**Etapas comparadas**		**Coeficiente 1**	**Coeficiente 2**	**Coeficiente 3**
Pré-clampeamento	Clampeamento	0,131	0,160	0,322
Pré-clampeamento	Pós-clampeamento	0,021	0,029	0,046
Clampeamento	Pós-clampeamento	0,016	0,029	0,046

**Figura 3:** Gráfico obtido com a monitorização por NIRS no voluntário 9, demonstrando, no eixo x, o tempo em minutos e, no eixo y, a saturação de oxigênio (SatO_2_; %) em lilás, a hemoglobina oxigenada (HbO; micro mol) em vermelho, a hemoglobina reduzida (HbR; micro mol) em azul e a hemoglobina total (HbT; micro mol) em verde; a área sombreada corresponde ao clampeamento.

Exemplo de gráfico (Figura 4) obtido em um dos doentes, correspondendo aos polinômios ajustados em que a etapa “pré-clampeamento” está representada em azul, o clampeamanto em vermelho e o “pós-clampeamento” em verde para cada uma das quatro variáveis utilizadas (SatO_2_, HbO, HbR, HbT).

**Figura 4:** Polinômios ajustados para o indivíduo 9, em que a etapa pré-clampeamento está representada em azul, o clampeamanto em vermelho e o pós-clampeamento em verde para cada uma das quatro variáveis utilizadas (SatO_2_, HbO, HbR, HbT).

## DISCUSSÃO

Desde a década de 1980 do século XX, foram publicados diversos estudos relacionando a utilização do infravermelho próximo como forma de monitorização da oxigenação tecidual em diversas condições clínicas. Porém, até o momento, nenhum artigo correlacionou a aplicação do infravermelho próximo durante a endarterectomia de artéria carótida por meio da monitorização contínua dos momentos pré, trans e pós-clampeamento, sendo a presente pesquisa a primeira a discorrer sobre esse tema.

Em termos teóricos e práticos, Murkin e Arango detectaram em seus trabalhos, por meio da NIRS, a isquemia em outros territórios, a exemplo de: esplâncnicos, renal e da coluna espinhal. Isso justifica a sensibilidade e aplicabilidade clínica do método na monitorização da perfusão cerebral contínua pela NIRS para detecção das alterações provocadas pelo tratamento cirúrgico no território carotídeo^3^.

Segundo Becquemin et al., existem alguns critérios que determinam a escolha do método cirúrgico a ser adotado nos pacientes portadores de estenose de carótida. A presença de doença coronariana, aortoilíaca ou de insuficiência renal, a idade (maior que 80 anos) e a história de neoplasia (possível tratamento com radioterapia) são exemplos de fatores de risco que têm influência direta na realização da endarterectomia de artéria carótida ou de angioplastia carotídea com stent^4^.

Todos os doentes da nossa amostra foram monitorizados no intraoperatório por meio de oximetria de pulso e capnografia, demonstrando estabilidade constante dos parâmetros durante o procedimento cirúrgico.

Quantos aos métodos de monitorização cerebral, diversas modalidades foram descritas na literatura, entretanto, não existe um método que demonstre, de maneira efetiva e segura, as alterações isquêmicas cerebrais. Portanto, a busca por métodos seguros e reprodutíveis que reduzam a morbimortalidade do ato operatório são constantes. Sloan afirma que, durante cirurgias vasculares em território carotídeo e cirurgias cardiovasculares, as técnicas de neuroimagem fornecem informações importantes, que podem ser vistas como implementadoras das técnicas cirúrgicas e, possivelmente, melhorar os resultados clínicos. Mas essas técnicas são imperfeitas, e as modalidades diagnósticas ainda não foram estabelecidas com precisão^5^.

O uso da NIRS tem demonstrado ser uma opção promissora nos próximos anos, porém, necessita de estudos clínicos amplos nas mais diversas áreas envolvendo isquemias teciduais. Fellahi et al. afirmaram que os valores da saturação de oxigênio são diferentes nos diversos leitos vasculares e que há uma diferença na tolerância a isquemia entre homens e mulheres^6^.

Os princípios de análise da NIRS consistem na aplicação de diferentes comprimentos de ondas, levando a diferenças qualitativas e quantitativas dos componentes moleculares de um tecido biológico. A captação do método depende dos efeitos de reflexão, dispersão e absorção.

A aplicabilidade da NIRS é muito ampla, exemplo disso pode ser observado no estudo realizado por Casati et al., em que, em pacientes idosos submetidos a cirurgias abdominais, a monitorização foi importante para direcionar o plano anestésico, reduzindo a exposição à isquemia, com menor efeito nas alterações cognitivas e no menor período de internação. Várias condições clínicas da prática médica diária possuem potencial para ocasionar alterações na oxigenação cerebral, levando ao risco de isquemia cerebral intraoperatória. Essas alterações no balanço da oxigenação cerebral identificáveis por meio de um método simples e eficaz têm o potencial de otimizar o plano anestésico para as reais necessidades de cada indivíduo para o principal órgão, o cérebro^7^.

Em nossa amostra, observamos que as medidas registradas pela NIRS diferem quanto às variáveis HbR e SatO_2_ nos momentos pré e pós-clampeamento em relação ao clampeamento. Isso reflete a isquemia tecidual cerebral detectada pelo método de maneira direta, com redução dos níveis de oxigênio tecidual e aumento dos níveis de CO_2_.

Mille et al.^8^, em 2004, em seu estudo sobre a monitorização com a NIRS durante a endarterectomia de carótida, objetivaram determinar quais pacientes possuíam uma boa colateralização da circulação cerebral durante o clampeamento da carótida por meio da determinação da porcentagem de decréscimo da saturação de oxigênio regional. Sugere-se que, com o decréscimo da saturação de oxigênio em relação ao valor pré-clampeamento, quando é menor ou igual a 20%, a isquemia e a hipoperfusão não são frequentes e o *shunt* não é necessário. Valores de decréscimo maiores que 20% nem sempre indicam uma complicação neurológica intraoperatória, mas podem servir como definidor de conduta^8^.

Yamamoto et al.^9^, em 2007, afirmaram que a hipoperfusão é um dos fatores que levam ao acidente vascular encefálico durante a endarterectomia de carótida no perioperatório. O *shunt* seletivo necessita de uma monitorização simples e sensível. Segundo os autores, a NIRS é um sistema de monitorização que pode ser usado durante a cirurgia e que reflete a oxigenação instantaneamente.

O método da NIRS e seu modo de aplicação são inéditos, o que justifica a pouca experiência da equipe, que optou por relatar sua experiência inicial e, em estudos subsequentes que estão em andamento, ampliar a amostra.

## Conclusões

A espectroscopia próxima a infravermelho (NIRS) é um método viável e aplicável de monitorização intracerebral, não invasivo e em tempo real, durante a endarterectomia carotídea.

Essa técnica é capaz de medir as mudanças dos níveis de saturação de oxigênio, hemoglobina total, hemoglobina reduzida e hemoglobina oxigenada durante os três momentos da endarterectomia de carótida (pré-, trans e pós-clampeamento).
